# Substituting maize and soybean meal with non-conventional feed ingredients: environmental trade-offs and resource-saving potential in China’s pig production

**DOI:** 10.1186/s40104-026-01458-z

**Published:** 2026-07-07

**Authors:** Qile Hu, Xue Bao, Nuo Xiao, Yuansen Yang, Changhua Lai, Lu Wang, Shuai Zhang

**Affiliations:** State Key Laboratory of Animal Nutrition and Feeding, Ministry of Agriculture and Rural Affairs Feed Industry Centre, College of Animal Science and Technology, China Agricultural University, Beijing, 100193 P. R. China

**Keywords:** Environmental impacts, Feed resource supply, Life-cycle assessment, Non-conventional feed ingredients, Pig production systems

## Abstract

**Background:**

China produces approximately one-quarter of the world’s grain with less than 9% of global arable land; however, more than half of this production is used for animal feed. Meanwhile, the feed system in China has long been highly dependent on conventional ingredients such as maize and soybean meal, resulting in a relatively homogeneous feed structure. Under the dual objectives of developing a diversified feed supply system and promoting sustainable livestock production, expanding the utilization of non-conventional feed ingredients (NCFIs) has become an important pathway. In this context, systematically quantifying their potential for greenhouse gas mitigation is essential for advancing the green and low-carbon transition of agriculture. Taking 1 kg live-weight gain in growing-finishing pigs as the functional unit (FU), three scenarios were designed to evaluate the life-cycle carbon footprint, nitrogen footprint, land use, and grain-saving effects of substituting maize and soybean meal with ten NCFIs in diets for 25–120 kg pigs across the complete “crop-pig-manure” chain.

**Results:**

Marked inter-type variability in environmental profiles was observed among the ten NCFIs: carbon footprint ranged from 2.46 to 2.84 kg CO_2_-eq/FU, nitrogen footprint from 60.49 to 82.02 g Nr/FU, and land use from 5.43 to 8.42 m^2^/FU. Substituting maize with rice bran and cassava reduced the carbon footprint, while cassava substitution additionally decreased nitrogen emissions and land use. All six NCFIs contributed to reductions in carbon footprint to varying extents, with peanut meal and palm kernel meal also leading to concurrent reductions in nitrogen footprint. Meanwhile, scenario-based simulations indicated that large-scale adoption of NCFI substitution could annually spare 27–90 million tons of maize or 15.84–30.98 million tons of soybean meal.

**Conclusion:**

Collectively, the NCFI strategy exhibits environmental trade-offs: while generally favorable for carbon reduction, the mitigation efficacy is feed ingredients-dependent, and adverse impacts on nitrogen emissions and land use must be managed. Cassava exhibits the greatest mitigation potential, whereas sorghum requires more careful formulation and supply chain management. These findings corroborate the substantial resource-replacement capacity of NCFIs and provide a quantitative basis for developing low-carbon, resource-efficient livestock systems.

**Supplementary Information:**

The online version contains supplementary material available at 10.1186/s40104-026-01458-z.

## Background

Livestock systems are distributed globally, and animal sourced foods (ASF) provide humans with 39% of their protein and 18% of their calorie intake according to the Food and Agriculture Organization [1]. The agricultural sector accounted for about 17% of global anthropogenic greenhouse gas emissions in 2019 [2]. With the continued expansion of livestock production, feed grain consumption has already exceeded grain for direct human consumption, and is now the main driver of total grain demand in China [3]. In addition, the production of ASF occupies 70% of global cropland [4].

In China, the feed supply system exhibits a pronounced reliance on external resources. Soybean imports, which serve as the primary source of soybean meal, have consistently exceeded 90 million tons in recent years, reaching approximately 105 million tons in 2024 [5, 6]. In contrast, maize is mainly domestically produced, but imports increased markedly after 2020, peaking at 28.35 million tons in 2021 and subsequently fluctuating between 20 and 27 million tons [7, 8]. Without structural adjustments, China’s feed demand is projected to increase by approximately 31.73 million tons by 2030, including an additional 21.2 million tons of maize and 6.98 million tons of soybean meal. Furthermore, long-distance transportation of these feed ingredients contributes to additional energy consumption and associated emissions.

In 2024, China marketed 702 million pigs, maintaining its positions as the world’s largest pork producer. Large-scale operations (≥ 500 head/year) accounted for 70% of total animals slaughtered, an increase of two percentage points over 2023 [9]. Meanwhile, increasing geopolitical uncertainties and evolving global trade patterns have introduced additional risks to the feed supply chain. The high concentration of soybean imports from a limited number of exporting countries, together with fluctuations in maize imports, indicates a degree of vulnerability to external shocks [5, 6]. Under these circumstances, enhancing the diversification of feed resource supply and reducing reliance on key imported commodities are essential for improving the resilience and sustainability of China’s livestock production systems.

Dietary diversification strategies for pig production have emerged as a new research frontier, aiming to reformulate diets and improve protein-use efficiency while alleviating environmental pressure [10]. Non-conventional feed ingredients (NCFIs), defined as ingredients with limited current use or poorly characterized nutritional value, including non-conventional energy sources, plant and animal proteins, and food-processing by-products, are gaining attention because of their wide availability and variable nutrient profiles [11, 12]. Harnessing local NCFIs offer a practical route to expand feed supply.

However, divergent yields, processing, transportation and nitrogen-utilization pathways among NCFI sources can generate substantial greenhouse gas emissions and nitrogen losses. Focusing solely on substitution rates and nutritional equivalence risks shifting, rather than reducing, environmental burdens. Under China’s dual-carbon targets, a lack of cradle-to-farm-gate quantification of these life-cycle impacts may not only prevent the ecological optimization of feed ingredient substitution strategies, but also introduces systematic deviations in national agricultural mitigation pathways.

Therefore, this study aimed to quantify the environmental benefits of optimizing feed formulation by developing a cradle-to-farm-gate life-cycle assessment (LCA) model for growing-finishing pigs in China. Substitution ingredients and inclusion rates were specified according to the national Technical Scheme for Reducing and Replacing Maize and Soybean Meal in Pig and Poultry Feed [13]. Scenario analysis was employed to elucidate the environmental emission profiles of NCFIs when deployed at scale within the pig production systems in China.

## Materials and methods

### Study scope

Given the accelerating intensification of the sector, the present study focuses on commercially housed grower-finisher pigs (25–120 kg live weight) to quantify the potential of NCFIs to replace maize and soybean meal in diets. A life-cycle perspective was adopted, including feed crop cultivation and processing, transportation, animal housing, and manure management.

### Definition of scenarios

Following the “Technical Plan for Reducing Corn and Soybean Meal in Pig and Poultry Diets” proposed by the MOA [13], three dietary scenarios were established: one baseline and two alternatives. The alternative scenarios collectively comprised ten specific combinations of NCFI type and inclusion level (Table 1). Business-as-usual (BAU): a conventional maize-soybean-meal diet formulated on a metabolizable-energy (ME) system to satisfy the entire nutrient requirements of grower-finisher pigs (25–120 kg live weight) and no NCFIs were supplemented. Based on the International Feed Numbering (IFN) system (See Table S1), two substitution scenarios were designed: Scenario 1 (S1)-partial replacement of maize with non-conventional energy feed ingredients, and Scenario 2 (S2)-partial replacement of soybean meal with non-conventional protein feed ingredients.

**Table 1:** Detailed instructions for the settings of BAU, S1 and S2

Scenarios^a^	Alternative types and detailed description	Proportions of NCFIs
BAU	BAU was formulated with the most common maize (73.74%) and soybean meal (22%) diet in pig production based on metabolizable energy (ME) system, which meet nutritional needs of growing pigs at all stages from 25 to 120 kg	No additional NCFIs added
S1: Scenario of NCFIs replaced part of the maize ingredients	S1 contained 4 specific adjustments, with different NCFIs replacing a certain proportion of corn based on ME system	S1-1: Sorghum replaced 50% of maize
		S1-2: Barley replaced 50% of maize
		S1-3: Rice bran replaced 15% of maize
		S1-4: Cassava meal replaced 20% of maize
S2: Scenario of NCFIs replaced part of the soybean meal ingredients	S2 contained 6 specific adjustments, with different NCFIs replacing a certain proportion of soybean meal based on ME system	S2-1: Rapeseed meal addition ratio was 10%
		S2-2: Cottonseed meal addition ratio was 10%
		S2-3: Peanut meal addition ratio was 10%
		S2-4: Sunflower meal addition ratio was 10%
		S2-5: Sesame meal addition ratio was 10%
		S2-6: Palm kernel meal addition ratio was 5%

^a^To meet the nutritional requirements of pigs, different feed formulations under scenarios needed to be designed at different growing stages, and all diets were formulated based on the Nutrient Requirement of Swine in China [14] to ensure normal growth of pigs

### Dietary phases and formulation strategy

The Chinese National Standard “Nutrient Requirements of Swine” [14] divides grower-finisher pigs (25–120 kg) into four body-weight (BW) phases and provides corresponding average daily gain, daily feed intake, and nutrient specifications (Table S2). Diets were formulated on a metabolizable energy (ME) basis using the key performance indices given in the National Standard. To avoid increasing model complexity and propagating analytical error, a single nutrient matrix was adopted. Because nutrient requirements decline as BW increases, the most demanding 25–50 kg phase was used as the reference. This specification is sufficient to cover the requirements of heavier pigs without further adjustment. The dietary formulations and calculated nutrient levels for each scenario are given in Table 2.

**Table 2:** Ingredients and nutrient compositions of different diets of pig production with 4 scenarios and 15 adjustments included (as-fed basis)

Items	Scenarios										
	BAU	S1-1	S1-2	S1-3	S1-4	S2-1	S2-2	S2-3	S2-4	S2-5	S2-6
Ingredients, %											
Maize	73.74	36.35	38.22	64.91	57.78	70.45	72.51	73.55	68.56	72.24	70.37
Soybean meal (CP ≥ 46%)	22.00	21.00	19.00	20.00	23.50	14.50	11.50	11.50	16.00	13.00	20.60
Sorghum	-	36.87	-	-	-	-	-	-	-	-	-
Barley	-	-	36.87	-	-	-	-	-	-	-	-
Rice bran	-	-	-	11.06	-	-	-	-	-	-	-
Cassava meal	-	-	-	-	14.75	-	-	-	-	-	-
Rapeseed meal	-	-	-	-	-	10.00	-	-	-	-	-
Cottonseed meal	-	-	-	-	-	-	10.00	-	-	-	-
Peanut meal	-	-	-	-	-	-	-	10.00	-	-	-
Sunflower meal	-	-	-	-	-	-	-	-	10.00	-	-
Sesame meal	-	-	-	-	-	-	-	-	-	10.00	-
Palm kernel meal	-	-	-	-	-	-	-	-	-	-	5.00
Soybean oil	0.00	1.60	1.80	0.00	0.00	1.10	1.50	0.50	1.30	0.50	0.00
L-Lysine H_2_SO_4_	0.50	0.60	0.49	0.51	0.44	0.65	0.79	0.78	0.61	0.80	0.53
DL-Methionine	0.13	0.14	0.12	0.08	0.11	0.05	0.09	0.09	0.07	0.00	0.07
L-Threonine	0.17	0.10	0.08	0.10	0.09	0.12	0.16	0.16	0.11	0.12	0.09
L-Tryptophan	0.05	0.04	0.03	0.04	0.03	0.05	0.05	0.05	0.05	0.04	0.04
L-Valine	0.11	0.00	0.09	0.00	0.00	0.02	0.04	0.01	0.00	0.00	0.00
Monocalcium phosphate	1.70	1.70	1.70	1.70	1.70	1.50	1.60	1.60	1.70	1.70	1.70
Limestone	0.75	0.75	0.75	0.75	0.75	0.70	0.90	0.90	0.75	0.75	0.75
Salt	0.35	0.35	0.35	0.35	0.35	0.36	0.36	0.36	0.35	0.35	0.35
Vitamin and mineral premix^a^	0.50	0.50	0.50	0.50	0.50	0.50	0.50	0.50	0.50	0.50	0.50
Total	100.00	100.00	100.00	100.00	100.00	100.00	100.00	100.00	100.00	100.00	100.00
Calculated nutrient levels^b^, %											
ME^c^, MJ/kg	13.83	13.66	13.66	13.82	13.74	13.68	13.66	13.66	13.66	13.73	13.74
NE^d^, MJ/kg	10.65	10.51	10.47	10.64	10.55	10.68	10.76	10.73	10.59	10.61	10.55
CP	16.21	16.28	16.16	16.19	16.06	16.17	16.29	16.33	16.08	16.13	16.03
SID lysine^e^	0.98	0.98	0.97	0.97	0.97	0.98	0.97	0.97	0.97	0.97	0.97
SID methionine + Cysteine	0.60	0.56	0.56	0.56	0.55	0.56	0.56	0.55	0.55	0.55	0.55
SID threonine	0.68	0.61	0.60	0.60	0.60	0.60	0.60	0.60	0.61	0.61	0.61
SID tryptophan	0.18	0.18	0.17	0.17	0.18	0.18	0.18	0.17	0.18	0.18	0.18
SID valine	0.78	0.72	0.65	0.65	0.66	0.66	0.65	0.66	0.67	0.66	0.69
Calcium	0.64	0.65	0.63	0.64	0.67	0.64	0.66	0.66	0.65	0.77	0.64
Phosphorus	0.49	0.50	0.50	0.50	0.49	0.45	0.46	0.52	0.50	0.50	0.49

^a^Premix provided the following quantities per kilogram of diets: vitamin A as retinyl acetate, 8,250; vitamin D_3_ as cholecalciferol, 825; vitamin E as DL-alpha-tocopherol acetate, 40 IU; vitamin K_3_ as menadione nicotinamide bisulfite, 4 mg; vitamin B_12_, 25 μg; riboflavin, 5; pantothenic acid as DL-calcium pantothenate, 15; niacin, 35; choline chloride, 600; folacin, 2; thiamin as thiamine mononitrate, 1; pyridoxine as pyridoxine hydrochloride, 2; biotin, 4; Mn as MnO, 25; Fe as FeSO_4_•H_2_O, 80; Zn as ZnSO_4_, 100; Cu as CuSO_4_•5H_2_O, 50; I as KI, 0.5; Se as Na_2_SeO_3_, 0.15 mg

^b^All the dietary nutrient concentrations were re-calculated based on the diet formulation and the nutrient compositions of ingredients published in Nutrient Requirement of swine: GB/T 39235-2020 [14]

^c^*ME* Metabolizable energy

^d^*NE* Net energy

^e^*SID* Standardized ileal digestibility

### LCA modeling and environmental impacts

**Fig. 1:**
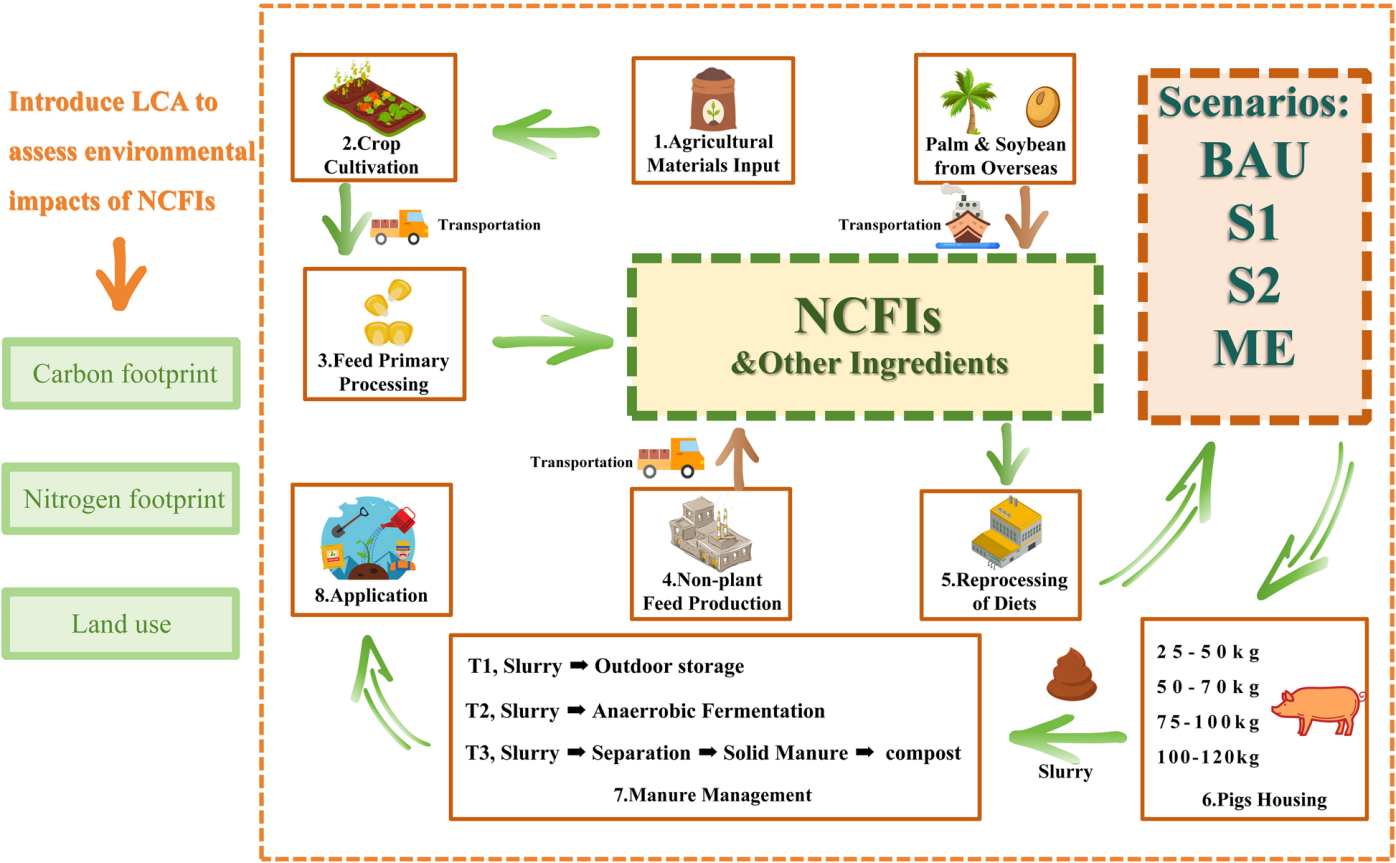
System boundaries of applying NCFIs in typical growing pig production system in China. The system with processes of agricultural materials input, feed crop cultivation, feed primary processing, reprocessing and transportation, pig housing and manure management. NCFIs, non-conventional feed ingredients; BAU, a business as usual scenario; S1, scenario 1, Scenario of NCFIs replaced part of the maize ingredients; S2, Scenario 2, Scenario of NCFIs replaced part of the soybean meal ingredients; Definitions of scenarios were detailed in Table 1. LCA, life cycle assessment; ME, metabolizable energy system; T1, treatment 1, representing that slurry or solid manure were stored outdoors and returned to the field; T2, treatment 2, representing that slurry or solid manure were returned to the field after anaerobic fermentation; T3, treatment 3, representing that slurry were separated and composted before returning to the field

Life-cycle assessment (LCA) is now the standard tool for multi-stage, multi-process supply-chain analysis and was used here to quantify national-scale environmental burdens of live-pig production. Following recent best-practice for pig systems [15], the production unit, attributional approach and system boundary were defined a priori. An attributional, “cradle-to-farm-gate” boundary (Fig. 1) was chosen with 1 kg live-weight at the farm gate as the functional unit. Economic allocation was applied to apportion the environmental load of soybean meal, rice bran and other co-products to feed ingredients according to their relative market prices.

The feed chain was disaggregated into imported (soybeans from Brazil, USA and Argentina; palm kernel meal from Malaysia and Indonesia) and domestic ingredients, covering cultivation, primary and secondary processing, and transportation (ocean freight plus 200 road transportation for imports, 400 for domestic crops, 1,000 km for micro-ingredients). Emission factors were compiled from Ecoinvent v3, official statistics, peer-reviewed literature, and the NUFER (NUtrient flows in Food chains, Environment and Resources use) model. Specifically, the NUFER model, originally developed by Ma et al. [16], was utilized as a well-established mass flow tool uniquely tailored to quantify nutrient losses and environmental emissions within China’s agricultural systems [17]. Based on these integrated sources, the crop phase included the manufacture and application of fertilizers and pesticides, direct field emissions of N_2_O, NH_3_ and NO_x_, leaching, run-off and erosion, together with straw return and biological N-fixation.

On-farm activities comprised energy use in housing, enteric CH_4_ and manure storage. Three manure-management scenarios were analyzed as: T1—raw slurry storage followed by land application; T2—anaerobic digestion (AD) before land spreading; T3—solid–liquid separation with composting of the solid fraction and AD of the liquid fraction prior to field application.

All greenhouse-gas emissions were converted to CO_2_-equivalents (CO_2_-eq) using the IPCC Sixth Assessment Report (AR6) 100-year global warming potentials (CO_2_ = 1, CH_4_ = 27, N_2_O = 273) [18]. Reactive nitrogen (Nr) losses were quantified as the sum of NH_3_, N_2_O, NO_x_ and total nitrogen discharged to water. Agricultural land occupation was calculated from the actual area required to produce each feed ingredient.

### Sensitivity analysis

The sensitivity check was applied to ensure the robustness of key parameters and the reliability of the underlying datasets. Seven groups of parameters were considered in this analysis. This study adopted normalized sensitivity coefficients in sensitivity analysis to analyze the carbon footprint and the nitrogen footprint affected by the above parameters. Detailed protocols were provided by Hu et al. [19].

## Results

### Basic data used for LCA analysis

**Fig. 2:**
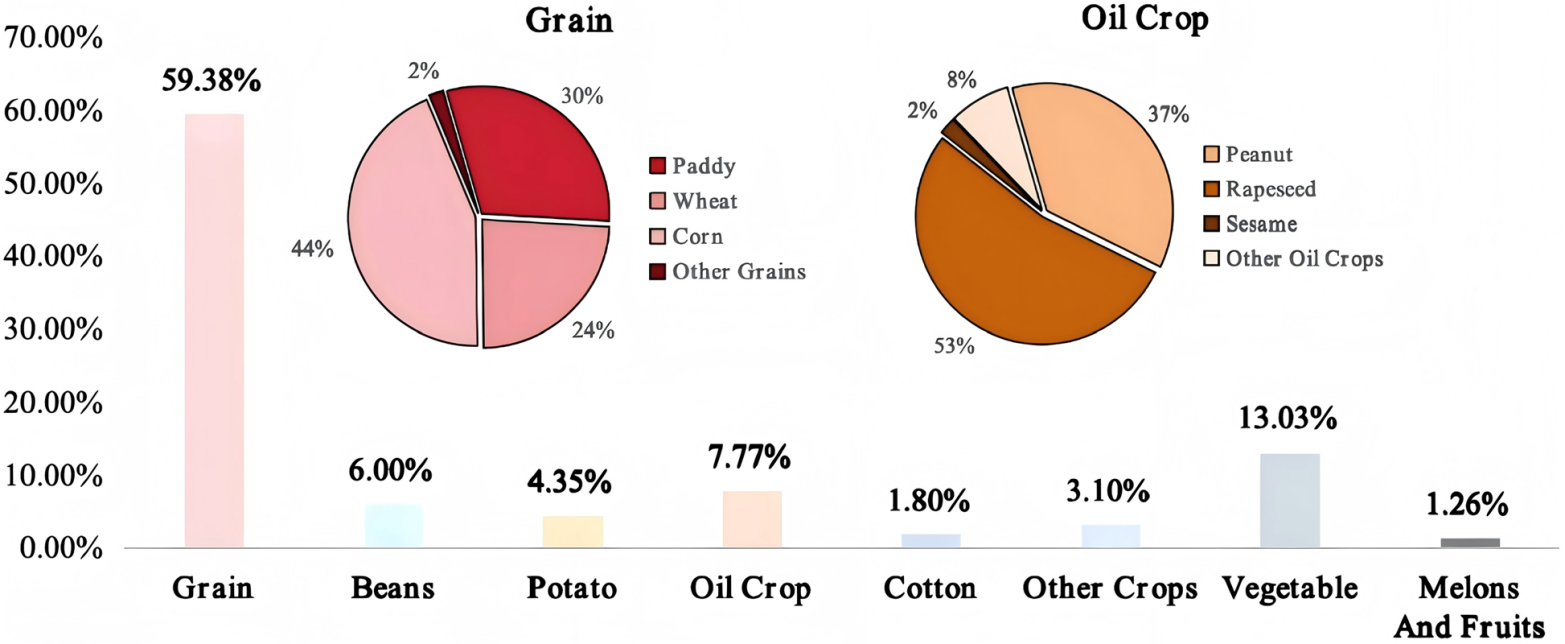
Planting structure of main crop types in China in 2021

The planting structure of China’s main crop types in 2021 was shown in Fig. 2. Global protein feed production and protein feed consumption of China in recent years were shown in Table 3, of which soybean meal alone accounted for 58.3%, with more than 90% imported [21]. Tianjin Ports serve as a major import hub for soybean and palm kernel raw materials in China, with overseas source ports primarily located in the Americas and Southeast Asia. Specifically, the American sources include Savannah (USA), Santos (Brazil), and Rosario (Argentina), with transport distances of approximately 9,795, 21,285, and 19,291 km, respectively. The Southeast Asian sources mainly include Manila and Semarang (Indonesia), with transport distances of approximately 3,100 and 5,776 km, respectively. Overall, the import routes are characterized by predominantly long-distance transoceanic transport, supplemented by shorter regional routes, indicating substantial spatial heterogeneity.

**Table 3:** Global protein feed production and protein feed consumption of China in recent years (million metric tons)

Item	Global production of protein feed^a^					Consumption of protein feed in China^b^
	2016	2017	2018	2019	2020	2022
Soybean meal	215.97	225.93	232.35	236.38	244.49	65.80
Rapeseed meal	38.61	38.80	39.53	39.47	39.41	11.60
Sunflower meal	16.51	19.34	19.89	21.13	22.15	3.30
Cottonseed meal	13.10	13.44	15.73	15.72	15.69	4.00
Palm kernel meal	8.18	8.91	9.77	10.11	9.96	-
Peanut meal	6.64	7.09	7.32	6.93	7.69	3.80
Fish meal	4.51	4.87	4.98	4.70	4.62	2.90
Copra meal	1.77	1.81	1.95	1.96	1.90	-
Other protein feed	-	-	-	-	-	21.50
Total	305.27	320.19	331.52	336.40	345.91	112.90

^a^Data from U.S. Department of Agriculture (USDA) [20]

^b^Data from Bureau of Animal Husbandry and Veterinary Medicine, Ministry of Agriculture and Rural Affairs of the People's Republic of China

### The carbon footprint of NCFIs

**Fig. 3:**
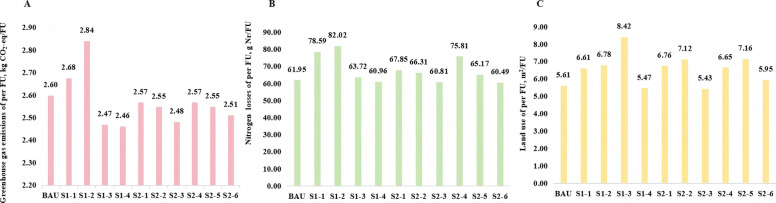
Environmental emissions associated with the production and use of NCFIs and other feed ingredients in the BAU and S1 and S2 scenarios throughout the life cycle of growing-finishing pigs. **A** Carbon footprint. **B** Nitrogen footprint. **C** Land use per FU. NCFIs, non-conventional feed ingredients; FU, functional unit; BAU, a business as usual scenario; S1, scenario 1, Scenario of NCFIs replaced part of the maize ingredients; S2, scenario 2, Scenario of NCFIs replaced part of the soybean meal ingredients. Definitions of scenarios were detailed in Table 1

**Fig. 4:**
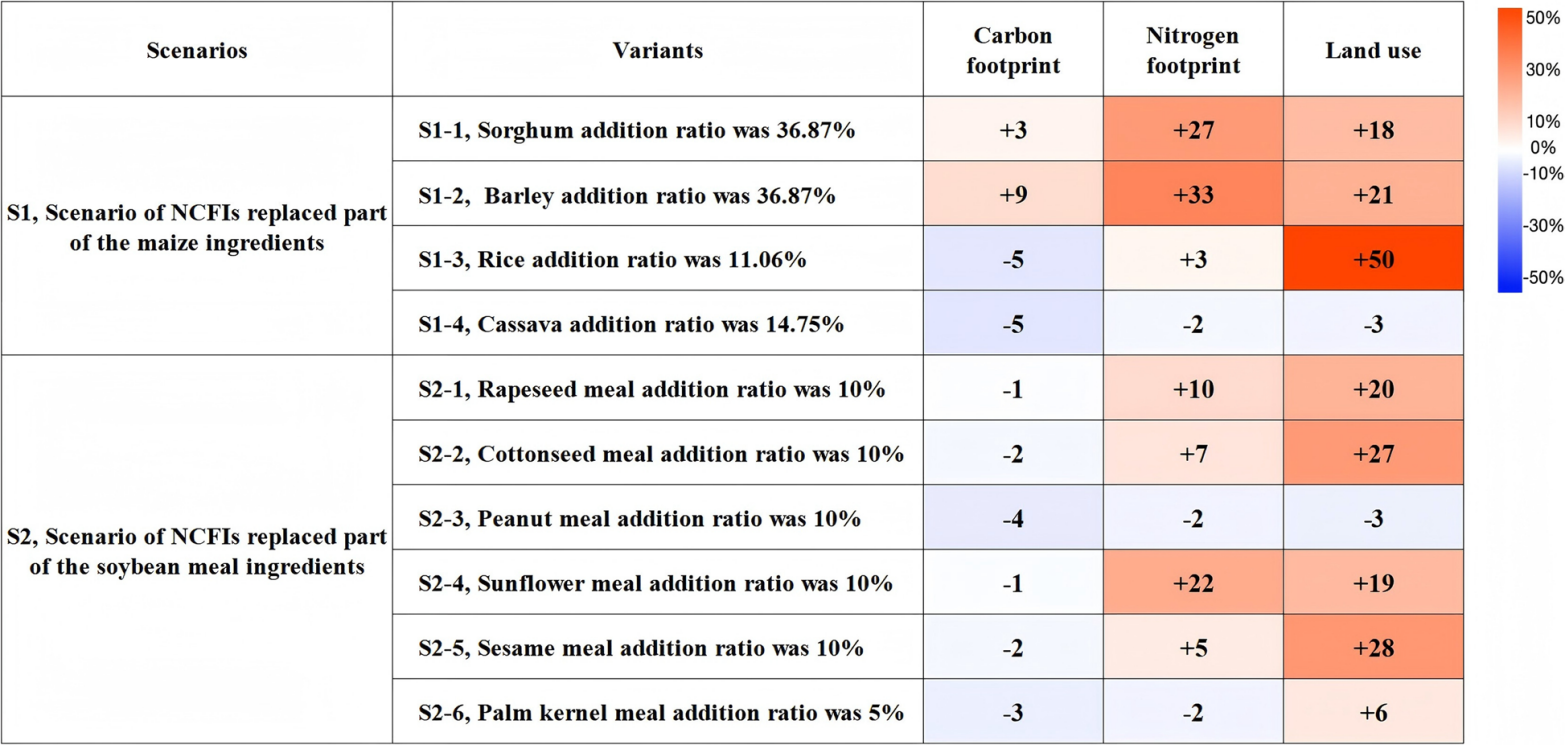
Relative changes (%) in carbon footprints, nitrogen footprints and land use following the implementation of different addition ratio of NCFI adjustments in scenarios, relative to the BAU baseline. Red colors indicated the environmental pressures increase, relative to the BAU baseline; Blue colors indicated the environmental pressures decrease, relative to the BAU baseline. BAU, a business as usual scenario; NCFIs, non-conventional feed ingredients; ME, the metabolizable energy system; S1, Scenario 1, Scenario of NCFIs replaced part of the maize ingredients; S2, Scenario 2, Scenario of NCFIs replaced part of the soybean meal ingredients

The cradle-to-farm-gate carbon footprints of pig production were shown in Fig. 3A. The carbon footprints for the four non-conventional energy ingredients scenarios (S1-1 to S1-4) and six non-conventional protein ingredients scenarios (S2-1 to S2-6) ranged from 2.46–2.84 and 2.48–2.57 kg CO_2_-eq/FU, respectively. Scenario of NCFIs replaced part of the soybean meal ingredients (S2) exhibited a lower mean (2.54 kg CO_2_-eq/FU) and narrower range of carbon footprints than scenario of NCFIs replaced part of the maize ingredients (S1, mean 2.61 kg CO_2_-eq/FU). The lowest greenhouse gas emissions were recorded for cassava addition (S1-4, 2.46 kg CO_2_-eq/FU) and peanut meal addition (S2-3, 2.48 kg CO_2_-eq/FU), whereas the highest greenhouse gas emissions corresponded to barley addition (S1-2, 2.84 kg CO_2_-eq/FU) and rapeseed meal addition (S2-1, 2.57 kg CO_2_-eq/FU). Compared with the BAU, rice bran addition (S1-3) and cassava addition (S1-4) delivered the greatest carbon-reduction potential, each lowering the system-wide carbon footprint by 4%, while sorghum addition (S1-1) and barley addition (S1-2) increased the carbon footprint to varying degrees (Fig. 4).

### The nitrogen footprint of NCFIs

The cradle-to-farm-gate nitrogen footprints of pig production were shown in Fig. 3B. Replacing maize with four non-conventional energy ingredients (S1-1 to S1-4) and soybean meal with six non-conventional protein ingredients (S2-1 to S2-6) yielded nitrogen footprints of 60.96–82.02 and 60.49–75.81 g Nr/FU, respectively. The mean Nr loss for the scenario of NCFIs replaced part of the soybean meal ingredients (S2, 66.07 g Nr/FU) was lower and less variable than that for the scenario of NCFIs replaced part of the maize ingredients (S1, 71.32 g Nr/FU). For the two scenarios, cassava addition (S1-4, 60.96 g Nr/FU) and palm-kernel meal addition (S2-6, 60.49 g Nr/FU) showed the lowest nitrogen footprint, whereas barley addition (S1-2, 82.02 g Nr/FU) and sunflower meal addition (S2-4, 75.81 g Nr/FU) showed the highest nitrogen footprint. Compared with BAU, cassava addition (S1-4), peanut meal addition (S2-3) and palm-kernel meal addition (S2-6) offered the greatest Nr mitigation potential, each reducing nitrogen footprint by 2%, while all other scenarios increased Nr losses to varying degrees (Fig. 4).

### The land use of NCFIs

The land use for pig-feed diets under the two categories of scenarios was shown in Fig. 3C. The land use in scenario of NCFIs replaced part of the maize ingredients (S1) ranged from 5.47 to 8.42 m^2^/FU, and the land use in scenario of NCFIs replaced part of the soybean meal ingredients (S2) ranged from 5.43 to 7.16 m^2^/FU. The mean land use values of S2 were slightly lower and less variable than that of S1 (6.81 m^2^/FU). Within each category, cassava addition (S1-4, 5.47 m^2^/FU) and peanut meal addition (S2-3, 5.43 m^2^/FU) delivered the lowest land occupation, whereas rice bran addition (S1-3, 8.42 m^2^/FU) and sesame meal addition (S2-5, 7.16 m^2^/FU) demonstrated the greatest land occupation. Relative to BAU, cassava addition (S1-4) and peanut meal addition (S2-3) provided the most pronounced land-use relief, each reducing land demand by 3%, while all other NCFI scenarios increased on-farm land use to varying extents (Fig. 4).

### The potential of NCFIs to substitute maize and soybean meal

According to the National Bureau of Statistics of China, national feed utilization in 2021 amounted to approximately 1.8 × 10^8^ t for maize and 6.9 × 10^7^ t for soybean meal [22]. In scenario of NCFIs replaced part of the maize ingredients (S1), dietary inclusion of 11.06%–36.87% non-conventional energy ingredients corresponds to a maize replacement rate of 15%–50%. Extrapolation under the idealized assumptions modeled here indicates that full-scale adoption could conserve 27–90 million tons of maize per year. In scenario of NCFIs replaced part of the soybean meal ingredients (S2), supplementation with 5%–10% non-conventional protein ingredients equates to replacing 23%–45% of soybean meal, yielding potential annual savings of 15.8–31.0 million tons of soybean meal.

### Sensitivity analysis

**Fig. 5:**
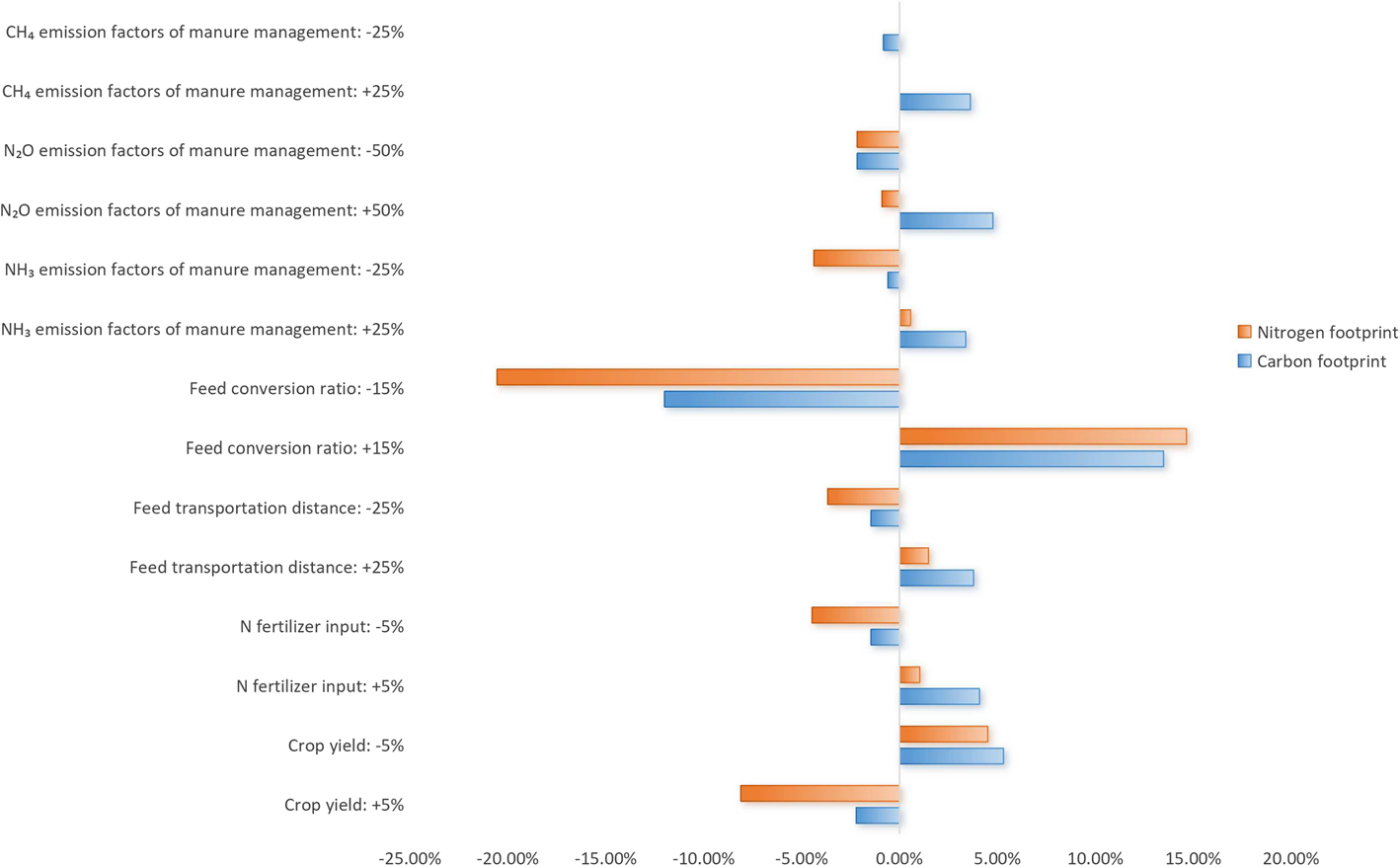
Effects of changing key parameters on the carbon footprint and the nitrogen footprint of pig production

The coefficient of variation for carbon and nitrogen footprints of pig production in China using ME system was presented in Fig. 5. Sensitivity analysis indicates that the feed conversion ratio (FCR) is the most sensitive parameter affecting the carbon and nitrogen footprints of pig production in China, with a ± 15% variation in FCR leading to fluctuations ranging from −21% to 15%. The impacts of variations in other factors (such as crop yield, nitrogen fertilizer use, transportation distance, and emissions of NH_3_, N_2_O, and CH_4_) on the carbon and nitrogen footprints all fall within the range of −5% to 5%.

## Discussion

### Environmental footprints of the NCFIs

The current study quantified the whole-life environmental burden of pig production in China from an animal-nutrition perspective. The standard NCFI diets partially replaced maize and soybean meal, and life-cycle carbon, nitrogen and land use were calculated for each feed formulation. In scenario of NCFIs replaced part of the maize ingredients (S1) and scenario of NCFIs replaced part of the soybean meal ingredients (S2), emissions did not always decline, consistent with earlier reports [23, 24]. The main driver is that feed production dominates the carbon footprint of pig farming [25–27]. Barley, the principal energy source in scenario of NCFIs replaced part of the maize ingredients (S1), requires more fertilizer and diesel than maize, so its cultivation emits more CO_2_. Transportation adds a further, noticeable share to feed-related emissions. Some authors argue that unconventional feedstuffs are often grown close to the farm and should therefore reduce transportation fuel use [28, 29]. In our study, both NCFIs and maize were assumed to be processed and fed on-site. However, because actual transport distances differ, the potential emission saving from utilizing local by-products were not captured. Nitrogen losses and land use followed the same trend. Dietary crude-fiber content varied across treatments. Zhang et al. [30] found that high-fiber diets can lower NH_3_ emissions during manure storage, but the precise relationship between dietary fiber and on-farm N loss remains unclear. As a result, our model omitted this factor.

Different NCFI sources exhibit markedly divergent emission profiles, with the disparity between unconventional energy feeds and unconventional protein feeds being particularly pronounced. This stems fundamentally from the fact that the replaced ingredients (maize and soybean meal) possess inherently distinct baseline emission levels. The cultivation of substitute cereals (barley) demands greater diesel and fertilizer inputs than maize cultivation, thereby elevating upstream CO_2_ emissions [31, 32]. An analogous issue prevailed in scenario of NCFIs replaced part of the soybean meal ingredients (S2). Rapeseed, cottonseed, and sunflower seed cultivated in China incur higher nitrogen losses during their growth cycle relative to soybean. Thus, the substitution of soybean meal with these oilseed meals augmented the nitrogen-loss component of the environmental footprint [33, 34].

Environmental impacts were evaluated for each growth stage; however, applying the nutrient specifications of the most demanding 25–50 kg phase across the entire growing–finishing period drives the environmental baseline to a conservative upper bound. Thus, heavier pigs received nutrients in excess of their requirements, driving up total nutrient inputs. This over-formulation explains why some NCFIs diets in our study exhibit a larger footprint than those reported elsewhere [26, 35]. Integrating multi-phase precision feeding strategies into future life-cycle models is expected to correct this baseline overestimation, thereby more fully unlocking the relative mitigation potential of NCFIs.

### The nutritional value of NCFIs

Most NCFIs exhibit inherent instability in their nutritional compositions, with variations significantly influenced by the origin, processing technology, and storage conditions of ingredients. Oilseed meals, such as cottonseed meal and rapeseed meal, contain anti-nutritional factors including gossypol and phytic acid, which directly limit their appropriate inclusion levels in diets [36, 37]. Cereal grains from different producing areas or processing enterprises show obvious differences in chemical compositions and bioavailability, leading to significant fluctuations in the digestibility of energy and nutrients when fed to growing pigs or sows [38, 39]. Mao et al. [40] concluded that mixed microbial and neutral-protease fermentation of rapeseed meal significantly improved growth performance and nutrient utilization in growing pigs. Shuai et al. [41] demonstrated that replacing part of the dietary ingredients with rapeseed meal co-fermented by Bacillus subtilis and exogenous enzymes effectively promoted nutrient digestion and absorption in finishing pigs. In summary, through advanced processing methods such as thermal treatment, fermentation, and enzymatic hydrolysis, it not only improves protein availability and energy utilization but also ensures the safety and stability of NCFIs in animal diets. Proper optimization of these processes enables large-scale application in sustainable livestock production systems.

### Sustainable utilization prospects of NCFIs

Substituting maize with selected NCFIs can alleviate the pressure on conventional feed resources and reduce competition for grain utilization. Most NCFIs are locally available by-products or region-specific feed resources with relatively low price elasticity. Therefore, their use may help mitigate cost shocks associated with price fluctuations in imported maize and soybean markets [42, 43]. In S1, substituting maize with rice bran and cassava reduced the carbon footprint, while cassava substitution additionally decreased nitrogen emissions and land use. In S2, all six NCFIs contributed to reductions in carbon footprint to varying extents, with peanut meal and palm kernel meal also leading to concurrent reductions in nitrogen footprint. In China, rice bran, as a by-product of rice processing, is primarily utilized as animal feed and for rice bran oil production, with approximately 60%–75% used in feed and 20%–30% for oil extraction [44, 45]. Cassava is mainly used as an energy feed ingredient and for starch processing, accounting for approximately 65%–80% and 20%–30% of total utilization, respectively [5, 46, 47]. Peanut meal is widely used in ruminant and aquaculture feed, with feed use accounting for approximately 60%–75%, while the remainder is used in food processing and export markets [5, 44]. Palm kernel meal, as a typical imported feed resource, is predominantly used in animal feed (> 90%) [46, 47]. Diets formulated with these NCFIs offer multiple advantages, including the development of a diversified feed supply system, mitigation of environmental impacts, and enhanced supply chain resilience. However, in other scenarios, although substitutions for maize and soybean meal are technically feasible, they are not always environmentally beneficial. For instance, non-conventional feed ingredients such as sorghum and barley were found to increase environmental footprints to varying degrees. These ingredient-specific responses are consistent with previous studies, which indicate that feed production dominates the carbon footprint of pig production systems, while transportation, crop inputs, and dietary nutrient balance jointly determine the overall environmental outcomes [23–27].

Yet the full mitigation and resource-saving potential remains unrealized, owing to both the nutritional risks of these ingredients and the inadequacy of current emission-calculation methods in reflecting on-farm conditions. Further research is required to unlock the complete value of NCFIs. Based on the emission characteristics of NCFIs, optimizing the pig production system from the perspective of low-carbon intensification can involve the combined use of feed ingredients by establishing a “carbon–nitrogen coupling coefficient” evaluation system. Zhang et al. [48] found that compared with a single-nutrient-level feeding strategy, the use of combined diets with low protein and high fiber achieves better results in reducing nitrogen loss from pig manure management systems. Huang et al. [49] pointed out in their research that prioritizing synergistic emission reduction combinations such as cassava and rapeseed meal (carbon footprint: 2.45 kg CO_2_-eq/FU; nitrogen footprint: 65.3 g Nr/FU) can avoid the diminishing marginal effect of single ingredient substitution. Optimization strategies should also extend to “end-of-pipe” manure management.

Precision feeding is a strong guarantee for the above nutritional strategies to mitigate environmental impacts [50]. Energy components account for 60%–70% of the total feed cost, making them a key consideration in feed formulation and precision feeding. For high-fiber feeds or NCFIs, the digestible energy (DE) or ME system tends to overestimate their true energy value. In contrast, the net energy (NE) system is more accurate [51]. The NE system in feed formulation has been proven to have the potential to reduce environmental impacts [19]. Multi-objective (MO) feed formulation is an emerging approach to balance economic cost, nutritional requirements, and environmental impacts in feed formulation, which can more comprehensively reflect the corresponding characteristics [52, 53]. The use of multi-objective feed formulation, including life cycle assessment data of feed ingredients, can reduce the environmental footprint of pork production in the United States, as demonstrated by Shurson et al. [54]. Therefore, NCFI-based diet schemes can be incorporated into the establishment of MO models and the prediction of relevant indicators to better evaluate the utilization value of foods not suitable for human consumption.

Livestock producers are recommended to utilize local NCFIs to develop circular agriculture models. Bi et al. [55] reported that promoting film-covered composting technology reduced NH_3_ volatilization from manure by 40%. Additionally, recycling biogas residues as fertilizers for NCFI crop cultivation forms a nutrient cycle, which can effectively reduce environmental emissions from the system. The spatial decoupling of crop and livestock production systems leads to low efficiency in the utilization of manure nutrients applied to cropland [56, 57]. Thus, crop growers miss out on “yield dividends”, meaning decoupled crop systems have lower yields [49]. Studies by Long et al. [23] and Tong et al. [58] discussed the application of intensive crop production strategies, reporting that technologies improving feed crop production and manure management can reduce carbon, phosphorus, and nitrogen losses. In layer production systems, He et al. [59] found that coupling crop and animal production systems using animal manure for field fertilization significantly reduced system nitrogen emissions. Circular agriculture can promote the development of the local agricultural economy. In this model, animal manure is effectively utilized, and producers can increase the unit yield of field crops through reasonable fertilization technologies and crop structure optimization. Meanwhile, using local feed sources can reduce the carbon footprint associated with fossil energy consumption during transportation [60]. Based on these studies, constructing an integrated crop-livestock model that encompasses crop cultivation, feed processing, and manure management is a crucial step. Strengthening scientific and technological support for grain production in China and advancing variety-improvement research for major crops such as maize, soybeans, rice, and wheat are equally important. On this basis, establishing a synergistic optimization system for varieties, cultivation practices, and resource management can serve as a key technical approach to improve resource-use efficiency at the source, reduce carbon-emission intensity per unit yield, and mitigate non-point-source pollution risks [13].

## Conclusion

Significant differences exist in the environmental emission characteristics across the full life cycle of pork production when NCFIs are used to replace maize or soybean meal. On the premise of meeting pigs’ nutritional requirements, the average carbon footprint, nitrogen footprint, and land use of pork production with four representative non-conventional energy feed ingredients were 2.61 kg CO_2_-eq/FU, 71.32 g Nr/FU, and 6.81 m^2^/FU, respectively. For six representative non-conventional protein feed ingredients, the corresponding average values were 2.54 kg CO_2_-eq/FU, 66.07 g Nr/FU, and 6.51 m^2^/FU, respectively. Cassava, peanut meal, and palm kernel meal exhibited considerable emission reduction potential. Based on the scenario analysis and idealized simulation calculations of this study, promoting NCFI substitution measures could save 27–90 million tons of maize or 15.84–30.98 million tons of soybean meal annually. Replacing maize with a single type of unconventional energy feed ingredient is likely to result in negative environmental impacts. In contrast, substituting soybean meal with a single type of NCFI can effectively reduce the carbon footprint, simultaneous mitigation in the nitrogen footprint and land use is not consistently guaranteed, highlighting inherent environmental trade-offs. In the future, research should employ multi-objective optimization techniques for formulating NCFIs, integrating environmental, nutritional, and economic dimensions. By scaling up the application of empirically validated NCFI combinations, this approach is expected to further enhance the sustainability of pork supply chains, playing a critical role in achieving China’s “dual carbon” goals (carbon peak and carbon neutrality) while diversifying the structure of feed resource supply.

## Supplementary Information

Additional file 1: Table S1. Eight classes of feeds based on composition and usage, each with a six-digit “International Feed Number ”. Table S2. Key performance indicators of a typical pig production system in China, as well as supporting references used in this study

## Data Availability

The data were presented in the main manuscript and supplemental materials.

## Abbreviations

AD: Anaerobic digestion
ASF: Animal sourced foods
BAU: Business-as-usual
BW: Body weight
CO_2_-eq: CO_2_-equivalents
DE: Digestible energy
FCR: Feed conversion ratio
FU: Functional unit
IFN: International Feed Numbering
LCA: Life-cycle assessment
ME: Metabolizable-energy
MO: Multi-objective
NCFIs: Non-conventional feed ingredients
NE: Net energy
Nr: Reactive nitrogen
