# Supplementary material for: Substituting maize and soybean meal with non-conventional feed ingredients: environmental trade-offs and resource-saving potential in China’s pig production
Source: J Anim Sci Biotechnol. 2026 Jul 7;17:141. doi: 10.1186/s40104-026-01458-z (PMC13340116; doi:10.1186/s40104-026-01458-z)
Supplement: Supplementary file 1 — Additional file 1: Table S1. Eight classes of feeds based on composition and usage, each with a six-digit “International Feed Number ”. Table S2. Key performance indicators of a typical pig production system in China, as well as supporting references used in this study [file 40104_2026_1458_MOESM1_ESM.docx]

**Supplementary Materials**

**Substituting maize and soybean meal with non-conventional feed ingredients: environmental trade-offs and resource-saving potential in China’s pig production**

Qile Hu^1†^, Xue Bao^1†^, Nuo Xiao^1^, Yuansen Yang^1^, Changhua Lai^1^, Lu Wang^1^, Shuai Zhang^1*^

^1^ State Key Laboratory of Animal Nutrition and Feeding, Ministry of Agriculture and Rural Affairs Feed Industry Centre, College of Animal Science and Technology, China Agricultural University, Beijing 100193, P. R. China.

*^*^* Corresponding authors. E-mails: Shuai Zhang, zhangshuai16@cau.edu.cn (S. Zhang)

^†^ These authors contributed equally to this work.

Carbon emissions were calculated based on greenhouse gas emissions including CO_2_, CH_4_ and N_2_O. Nitrogen emissions were calculated based on nitrogen flows and the Nr emission factors. The life cycle assessment (LCA) modelling and the calculation of carbon and nitrogen footprints follow the approach established in [1].

**1. Agricultural input and crop cultivation**

Emissions from feed crop production and processing take into account the following processes: production of agricultural products (such as fertilizers, pesticides, diesel and electricity for agricultural machinery), and cultivation of feed crops. This study considers soybeans and palm kernels (100% of palm kernel imported) produced abroad (soybeans: Brazil, the United States, and Argentina; palm kernel: Malaysia and Indonesia) consistent with China’s actual imports in 2019 (i.e., 80.6% of soybeans imported) [2]. We calculated the production and direct energy use of domestic feed crops based on the "National Agricultural Product Revenue and Cost Data Compilation" [3]. According to the actual production of various countries, the data of the relevant research and the data of the Ecoinvent Database Version 3 were used [4–9].

**2. Feed primary processing**

This stage included production of non-plant feeds, primary processing and reprocessing of feed ingredients. For the production of non-plant feed ingredients, LCAs were quantified to quantify the emissions: this study was concentrated in the feed composition (salt, phosphate, limestone and amino acids in the formula) [10,11]. Emissions derived from the second stage of feed processing (reprocessing) mainly involved in the following steps: crushing, mixing, expanding, and pelleting. The data used here is to treat a ton of pig feed for a total of 90 kWh of electricity. This is based on a survey estimate in China [12].

**3. Transportation**

Land transportation and ship transportation were considered in this part. Assuming that the initial crops were processed nearby, and it was not transported during this time. Assuming that the truck from the grain planting area to the feed factory was 200 kilometers. Imported feed was assumed that they entered China from Tianjin Port. The shipping distance between the country can be calculated in Netpas Distance [13].

**4. Pig housing and manure management**

The pig houses and feces management were detailed to the production of pigs, indoor and outdoor storage, stool treatment and application in order to be more accurate and comprehensive. The manure treatment methods were divided into three types: slurry or solid manure were stored outdoors and returned to the field; slurry or solid manure were returned to the field after anaerobic fermentation; slurry were separated and composted before returning to the field. There are various treatment methods for livestock and poultry manure. For indoor storage, solid floors are the predominant option, accounting for 62%, followed by slatted floors at 38%. In outdoor storage, impermeable surfaces without coverage make up 70%, while permeable surfaces without coverage and impermeable surfaces with coverage account for 10% and 20%, respectively. Regarding manure treatment, direct discharge accounts for 10%, anaerobic digestion for 6.5%, composting for 18.8%, and application for 60% [12,14–19].

**5. Economic allocation**

Economic allocation is allocated based on the relative economic value of major products and by-products on environmental impacts [20]. The allocation factors (AFs) of different feed ingredients, including soybean meal, rapeseed meal, peanut meal, cottonseed meal, rice bran, sunflower meal, sesame meal and palm kernel meal, used for calculation in the current study were 64%, 34%, 16%, 20%, 5%, 35%, 11% and 43%, respectively [21].

**Supplementary Tables**

**Table S1** Eight classes of feeds based on composition and usage, each with a six-digit “International Feed Number (IFN)”

| **Code** | **IFN^1^** | **Class Description** | |
| --- | --- | --- | --- |
| 1 | 1-00-000 | Dry forages and roughages | Hay; straw; fodder (aerial part); stover (aerial part without ears, without husks or aerial part without heads); other products with more than 18 percent crude fibre (dry basis); HULLS |
|  |  |  | This class includes all forages and roughages cut and cured. Forages or roughages are low in net energy per unit weight, usually because of the high fibre content. Thus, such products as SEED COATS, PODS, rice BRAN, etc. are included in this group |
| 2 | 2-00-000 | Pasture, range plants, and forages fed green | Included in this group are all forage feeds either not cut (including feeds cured on the stem) or cut and fed fresh |
| 3 | 3-00-000 | Silages | This class includes only ensiled forages (MAIZE, ALFALFA, GRASS, etc.), but not ensiled FISH, GRAIN, ROOTS and TUBERS |
| 4 | 4-00-000 | Energy feeds | Included in this group are products with less than 20 percent protein (dry basis) and less than 18 percent crude fibre (dry basis) as, for example, FISH, GRAIN, mill by-products |
| 5 | 5-00-000 | Protein supplements | This class includes products which contain 20 percent or more of protein (dry basis) from animal origin (including ensiled products) as well as oil meals, GLUTEN, et |
| 6 | 6-00-000 | Mineral supplements | - |
| 7 | 7-00-000 | Vitamin supplements (including ensiled yeast) | - |
| 8 | 8-00-000 | Additives | This class includes further feed supplements as antibiotics, colouring materials, flavours, hormones and medicants |

^1^IFN = International Feed Number. A six-digit IFN was assigned to each feed description. The first digit of this IFN denoted the class of feed.

Data sourced from FAO [22].

**Table S2** Key performance indicators of a typical pig production system in China

| Indicators | Value |
| --- | --- |
| Piglets per sow per year ^a^ | 23.96 |
| Replacement rate of sow, % ^a^ | 39.16 |
| Alive rate of weaning piglets, % ^a^ | 93.04 |
| ADG from 3~8 kg, g ^b^ | 220 |
| ADFI from 3~8 kg, g ^b^ | 290 |
| ADG from 8~25 kg, g ^b^ | 500 |
| ADFI from 8~25 kg, g ^b^ | 835 |
| ADG from 25~50 kg, g ^b^ | 750 |
| ADFI from 25~50 kg, g ^b^ | 1600 |
| ADG from 50~75 kg, g ^b^ | 880 |
| ADFI from 50~75 kg, g ^b^ | 2250 |
| ADG from 75~100 kg, g ^b^ | 900 |
| ADFI from 75~100 kg, g ^b^ | 2710 |
| ADG from 100~120 kg, g ^b^ | 860 |
| ADFI from 100~120 kg, g ^b^ | 2900 |
| Feed to gain ratio from 3~120 kg ^b^ | 2.52 |

^a^ Average value from 2016-2019. Data were from Gao et al. [23].

^b^ ADG, average daily gain; ADFI, average daily feed intake. Data were from Nutrient requirements of swine: GB/T 39235-2020 [24].

**Supplementary References**

1. Hu QL, Shi HW, Wang L, Wang L, Hou Y, Wang HL, et al. Mitigating environmental impacts using net energy system in feed formulation in China's pig production. Sci Total Environ. 2023;856:159103. https://doi.org/10.1016/j.scitotenv.2022.159103.

2. Ministry of Agriculture and Rural Affairs of the People's Republic of China. China Agricultural Trade Development Report. Beijing: China Agriculture Press; 2020. <http://www.moa.gov.cn/.> Accessed 12 Aug 2025. (In Chinese).

3. National Development and Reform Commission of China. National Data Compilation of Revenue and Cost of Agricultural Products. Beijing: China Statistics Press; 2019. <https://www.ndrc.gov.cn/.> Accessed 12 Aug 2025. (In Chinese).

4. Dalgaard R, Schmidt J, Halberg N, Christensen P, Thrane M, Pengue WA. LCA of soybean meal. Int J Life Cycle Assess. 2008;13:240-254. <https://doi.org/10.1065/lca2007.06.342.>

5. Sieverding HL, Bailey LM, Hengen TJ, Clay DE, Stone JJ. Meta-analysis of soybean-based biodiesel. J Environ Qual. 2015;44:1038-1048. <https://doi.org/10.2134/jeq2014.07.0320.>

6. Silva VPD, van der Werf HMG, Spies A, Soares SR. Variability in environmental impacts of Brazilian soybean according to crop production and transport scenarios. J Environ Manage. 2010;91:1831-1839. <https://doi.org/10.1016/j.jenvman.2010.04.001.>

7. Silva VPD, van der Werf HMG, Soares SR, Corson MS. Environmental impacts of French and Brazilian broiler chicken production scenarios: an LCA approach. J Environ Manage. 2014;133:222-231. <https://doi.org/10.1016/j.jenvman.2013.12.011.>

8. Wernet G, Bauer C, Steubing B, Reinhard J, Moreno RE, Weidema B. The ecoinvent database version 3 (part I): overview and methodology. Int J Life Cycle Assess. 2016;21:1218-1230. <https://doi.org/10.1007/s11367-016-1087-8.>

9. De Rosa M, Schmidt J, Pasang H. Industry-driven mitigation measures can reduce GHG emissions of palm oil. J Clean Prod. 2022;365:132565. <https://doi.org/10.1016/j.jclepro.2022.132565.>

10. Mosnier E, van der Werf HMG, Boissy J, Dourmad JY. Evaluation of the environmental implications of the incorporation of feed-use amino acids in the manufacturing of pig and broiler feeds using life cycle assessment. Animal. 2011;5:1972-1983. <https://doi.org/10.1017/S1751731111001078.>

11. Garcia-Launay F, van der Werf HMG, Nguyen TTH, Le Tutour L, Dourmad JY. Evaluation of the environmental implications of the incorporation of feed-use amino acids in pig production using life cycle assessment. Livest Sci. 2014;161:158-175. <https://doi.org/10.1016/j.livsci.2013.11.027.>

12. Liu D, Wang FH, Ma L, Ma WQ, Zhang FS. Estimation of NH3 emission factor for pig manure in China. Agric Eng. 2008;4:218-224. <https://kns.cnki.net/.> Accessed 15 Sept 2025. (In Chinese).

13. Netpas Distance. Netpas Distance software. 2019. <https://www.netpas.net/.> Accessed 15 Sept 2025.

14. Long WT, Wang HY, Hou Y, Chadwick D, Ma YF, Cui ZL, et al. Mitigation of multiple environmental footprints for China's pig production using different land use strategies. Environ Sci Technol. 2021;55:4440-4451. <https://doi.org/10.1021/acs.est.0c08359.>

15. Wei S, Bai ZH, Chadwick D, Hou Y, Qin W, Zhao ZQ, et al. Greenhouse gas and ammonia emissions and mitigation options from livestock production in peri-urban agriculture: Beijing - a case study. J Clean Prod. 2018;178:515-525. <https://doi.org/10.1016/j.jclepro.2017.12.257.>

16. Wu H, Wang S, Gao L, Zhang L, Yuan Z, Fan T, et al. Nutrient-derived environmental impacts in Chinese agriculture during 1978–2015. J Environ Manage. 2018;217:762-774. <https://doi.org/10.1016/j.jenvman.2018.04.002.>

17. Qu MS. Characteristics of nutrients in biogas slurry and the application effects in arable fields of Beijing suburb. Beijing: China Agricultural University; 2012. <https://kns.cnki.net/.> Accessed 15 Sept 2025. (In Chinese).

18. Xuan M, Xu ZC, Wu GY. Analysis of utilization of fecal resources in large-scale livestock and poultry breeding in China. J Agric Resour Environ. 2018;35:126-132. <https://doi.org/10.13254/j.jare.2017.0223.> (In Chinese).

19. Jia W. Studies on the evaluation of nutrient resources derived from manure and optimized utilization in arable land of China. Beijing: China Agricultural University; 2014. <https://kns.cnki.net/.> Accessed 15 Sept 2025. (In Chinese).

20. De Vries M, de Boer IJM. Comparing environmental impacts for livestock products: a review of life cycle assessments. Livest Sci. 2010;128:1-11. <https://doi.org/10.1016/j.livsci.2009.11.007.>

21. China National Grain and Oils Information Center. China National Grain and Oils Information Center official website. 2019. <http://www.grainoil.com.cn/.> Accessed 12 Aug 2025.

22. Food and Agriculture Organization of the United Nations. Fish Feed Technology. Rome: FAO; 1980. <https://www.fao.org/3/X5738E/x5738e00.htm#Contents.> Accessed 12 Aug 2025.

23. Gao KG, Wang L, Hu SL, Yang XF, Jiang ZY. Investigation and analysis on reproductive performance of sows in scaled pig farms in China. China Anim Husb Vet Med. 2019;46(1):155-157. <https://kns.cnki.net/.> Accessed 15 Sept 2025. (In Chinese).

24. National Technical Committee on Animal Husbandry of Standardization Administration of China. Nutrient requirements of swine: GB/T 39235-2020. Beijing: Standards Press of China; 2020.
